# Genetics of *PICALM* Expression and Alzheimer's Disease

**DOI:** 10.1371/journal.pone.0091242

**Published:** 2014-03-11

**Authors:** Ishita Parikh, David W. Fardo, Steven Estus

**Affiliations:** 1 Sanders-Brown Center on Aging, Department of Physiology, University of Kentucky, Lexington, Kentucky, United States of America; 2 Sanders-Brown Center on Aging, Department of Biostatistics, University of Kentucky, Lexington, Kentucky, United States of America; University of Florida, United States of America

## Abstract

Novel Alzheimer's disease (AD) risk factors have been identified by genome-wide association studies. Elucidating the mechanism underlying these factors is critical to the validation process and, by identifying rate-limiting steps in AD risk, may yield novel therapeutic targets. Here, we evaluated the association between the AD-associated polymorphism rs3851179 near *PICALM*, which encodes a clathrin-coated pit accessory protein. Immunostaining established that PICALM is expressed predominately in microvessels in human brain. Consistent with this finding, *PICALM* mRNA expression correlated with expression of the endothelial genes *vWF* and *CD31*. Additionally, we found that PICALM expression was modestly increased with the rs3851179A AD-protective allele. Analysis of *PICALM* isoforms found several isoforms lacking exons encoding elements previously identified as critical to PICALM function. Increased expression of the common isoform lacking exon 13 was also associated with the rs3851179A protective allele; this association was not apparent when this isoform was compared with total *PICALM* expression, indicating that the SNP is associated with total *PICALM* expression and not this isoform per se. Interestingly, *PICALM* lacking exons 2–4 was not associated with rs3851179 but was associated with rs592297, which is located in exon 5. Thus, our primary findings are that multiple *PICALM* isoforms are expressed in the human brain, that PICALM is robustly expressed in microvessels, and that expression of total *PICALM* is modestly correlated with the AD-associated SNP rs3851179. We interpret these results as suggesting that increased *PICALM* expression in the microvasculature may reduce AD risk.

## Introduction

Alzheimer's disease (AD) is a devastating disease marked by cognition and memory decline, affecting the elderly population. Twin and family-based studies suggest that sporadic late onset AD risk is genetically linked [1], [2]. Recent genome wide association studies (GWAS) have identified loci of genetic variance, single nucleotide polymorphisms (SNP)s, that are associated with AD risk [3]–. Elucidating the mechanism of action of these SNPs validates the SNP as an AD risk factor and may identify novel AD pathways. Additionally, since steps in AD pathways that are modulated by genetics may be susceptible to pharmacologic manipulation, identifying the actions of AD-associated SNPs may lead to robust new pharmacologic targets.

One of these SNPs is near the gene *PICALM* (phosphatidylinositol binding clathrin assembly protein) which is involved in endocytosis. The primary AD-associated SNP is rs3851179 [9]–[11], which lies approximately 80 kb 5′ of *PICALM*. *PICALM* itself is encoded by 21 exons, several of which are variably spliced [12]. Here, we sought to elucidate how rs3851179 alters *PICALM* expression or splicing to modulate AD risk. We report that PICALM is expressed robustly in microvessels and moderately in other cell types. Rs3851179 was modestly associated with total *PICALM* expression as well as the major *PICALM* isoform lacking exon 13. In contrast, the expression of rare *PICALM* isoforms lacking exons 2, 2–4, or 18–19 was not associated with rs3851179. We interpret our results as suggesting that the PICALM is robustly expressed in microvessels and that the protective rs3851179A allele is associated with modestly increased *PICALM* expression. We speculate that increased microvessel *PICALM* reduces AD risk, perhaps by facilitating Aβ clearance from the brain through enhanced translocation across the blood brain barrier.

## Materials and Methods

### Ethics Statement

The work described here was performed with approval from the University of Kentucky Institutional Review Board.

### Tissue samples

The RNA and DNA samples for this study were from de-identified AD and non-AD autopsy samples. Anterior cingulate specimens *were* provided by the University of Kentucky AD Center Neuropathology Core and have been described previously [13], [14]. A total of 52 brain samples, 28 male and 24 female, were used for this study. All of the non-AD individuals were cognitively intact at their last visit (MMSE of 27.9±3.4 (mean ± SD)). AD individuals were demented (MMSE of 12.2±8.3). For the AD autopsy samples, the average age at death and postmortem interval was 82.9±6.4 years (mean ± SD) and 3.4±0.7 hrs, respectively. For the non-AD samples, the average age at death and postmortem interval was 82.3±8.7 years (mean ± SD) and 2.8±0.8 hrs, respectively. By NIARI neuropathology criteria, the non-AD individuals included 21 samples with a score of no-low likelihood of AD, and 6 with intermediate. The AD samples were uniformly high-likelihood of AD. RNA was prepared by the method of Chomczynski and Sacchi [15] and converted to cDNA with random hexamers and Superscript II, as described previously [13]. Although RNA integrity analyses were not performed prior to reverse transcription, others have demonstrated that for qPCR with short amplicons, normalized expression differences are comparable between samples with moderate RNA degradation and those with high integrity RNA [16].

### PICALM immunostaining

Paraffin-embedded anterior cingulate tissue sections (5 μm thick) were rehydrated, underwent heat-induced antigen retrieval in citrate buffer (pH 6.0) and were quenched in 0.3% H_2_O_2_. Sections were immersed in 10% rabbit serum in Tris-buffered saline followed by an overnight incubation in anti-CALM (sc-6433, Santa-Cruz; 1∶400 dilution). After thorough rinsing in Tris-buffered saline, sections were incubated in biotinylated secondary antibody for 1 h, rinsed, incubated in ABC reagent (Vector) for 1 h, developed in Nova Red chromagen (Vector) and counterstained with Hematoxylin.

### Identification of PICALM Splice Variants in Human Brain

Screening for *PICALM* splice variants was performed on a pool of cDNA samples from five AD and five non-AD individuals. This cDNA pool was amplified by PCR by using forward and reverse primers designed to produce overlapping products; this enabled evaluation of splicing efficiency of each internal exon (Table 1). The identity of splice variants was determined by sequencing. To estimate the distribution of these splice variants, exon 12–20 PCR products from three rs3851179 homozygous minor (A/A) and three homozygous major (G/G) individuals were TA-cloned (Invitrogen) and 847 random clones were sequenced. For this work, thirty cycles of PCR (Platinum Taq, Invitrogen) were performed by using primers corresponding to exons 12 and 20 (Table 1). PCR conditions were 94° for 15 seconds, 60° for 15 seconds, and 72° for 60 seconds (Veriti 96-Well Thermal Cycler, Life Tech). PCR was conducted using approximately 30 ng of cDNA template. After PCR, samples were cloned into pcDNA2.1 according to the manufacturer's instructions (TA-Cloning Kit, Invitrogen) and sequenced.

**Table 1:** PCR Primers.

Target	Name		Sequence (5′-3′)
Exons 1–5	1F	Sense	CTGACGGACCGAATCACTG
	5R	Antisense	TCAAGAAGTGCATCCATCTGA
Exons 3–9	3F	Sense	TGGCTTCAAGAAACACGTTG
	9R	Antisense	GCTTGCAGCTGTAGAATCTTTG
Exons 7–12	7F	Sense	TGAAAAAGAACCAATGCAAAGA
	12R	Antisense	CCCCATGTACTTGCTACCTGA
Exons 10–14	10F	Sense	CTTTCCAATGCAGTGTCTTCC
	14R	Antisense	CCCCAGAATCTACTACAATAACATTTG
Exons 12–17	12F	Sense	GCCCAATGATCTGCTTGATT
	17R	Antisense	CATTGTTGCAGCATTCCAAG
Exons 15–20	15F	Sense	GCTTTGATGAACTAGGTGGACTT
	20R	Antisense	GCAGTTTGGATTTTGCTGGA
Total PICALM	9	Sense	ACAGGCCCCTAGCAGTCTTC
	10	Antisense	TGCTTTTCCCTTTCATCCAC
D13	11	Sense	TGCAGCCTCTCCTGTATCCACCT
	12–14 Junction	Antisense	GGAGAAGGAGTGAATCCTCCC
D18–19	17	Sense	TGGAGTCAACCAGGTGAAAA
	17–20 Junction	Antisense	CATTTGTGGAGGCATTGTTG
D2	1	Sense	GAGGAGCTGCAGAGATGTCC
	1–3 Junction	Antisense	TACTGAATAAAACGAGTCCAGGTG
D2–4	1–5 Junction	Sense	AAAGCACCTGGACTGGCTGA
	6	Antisense	GGCAGCATTTATTACCCCATT
PECAM1	CD31F	Sense	ATTGCAGTGGTTATCATCGGAGT
	CD31R	Antisense	CTCGTTGTTGGAGTTCAGAAGTGG
VWF	vWF F	Sense	CGGCTTGCACCATTCAGCTA
	vWF R	Antisense	TGCAGAAGTGAGTATCACAGCCATC
Exon12–20	12	Sense	GCCCAATGATCTGCTTGATT
	20	Antisense	TTGGTTGCGTCATTACAGGA

PCR primers used for screening splice variants, cloning, qPCR and sequencing.

### Quantitation of PICALM Expression

Total *PICALM* expression was quantified by qPCR using primers corresponding to sequences within the constitutively present exons 9 and 10 (Table 1); *PICALM* isoforms lacking exon 2, exons 2–4, 13 or 18–19 were quantified similarly (Table 1). As no single Ensembl transcript incorporates each of the exons that we identify here, note that our exon designations are derived from ENST00000393346 for exons 1–16. Exons 17–21 correspond to the final five exons within ENST00000532317. PCR was conducted using an initial 2-minute incubation at 95°, followed by cycles of 10 seconds at 95°, 20 seconds at 60°, and 20 seconds at 72°. The 20 μL reactions contained 1 μM of each primer, 1x PerfeCTa SYBR Green Super Mix (Quanta Biosciences), and 30 ng cDNA. Experimental samples were amplified in parallel with serially diluted standards that were generated by PCR of cDNA using the indicated primers followed by purification and quantitation by UV absorbance. Results from samples were compared relative to the standard curve to calculate copy number in each sample. Real time assays were performed at least twice and the average copy number used for data analyses. Since *PICALM* was expressed in microvessels, neurons and astrocytes, we wished to compare *PICALM* expression to that of genes specific to these cell types. Hence, we also quantified two microvessel-specific mRNAs, *CD31* and von Willebrand Factor (*vWF*), neuron-specific mRNA *SYP* and astrocyte-specific *GFAP*. [17], [18]. The copy number for each mRNA was then normalized to the geometric mean of reference genes *RPL32* and *EIF4H*, previously quantified in this sample set [13], [14]. The linear regression statistical model used to analyze the data included the geometric mean of *CD31* and *vWF* (microvessel mRNA), *GFAP, SYP*, AD status and the number of rs3851179 minor alleles (SPSS version 21).

## Results

**Figure 1:**
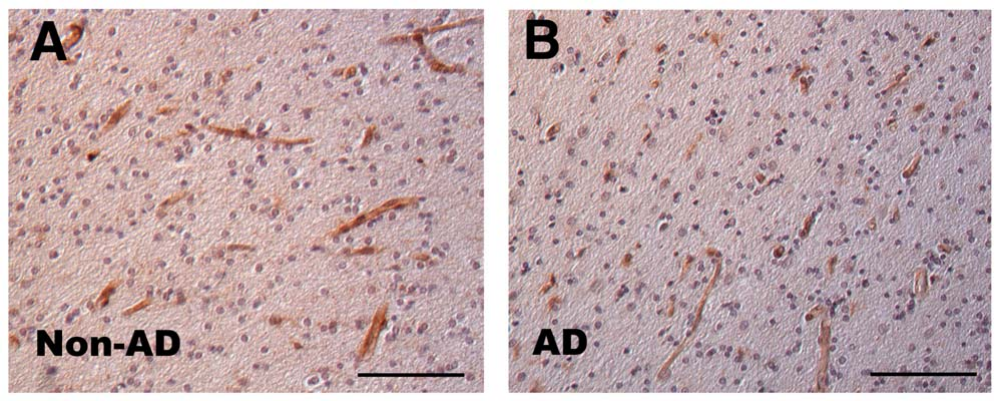
PICALM immunohistochemistry in human brain. Human anterior cingulate was immunostained with anti-CALM antibody, revealing robust microvessel labeling (bar = 100 μm).

To begin to evaluate the role of *PICALM* in AD, we localized PICALM expression in human brain by performing immunohistochemistry. We used an antibody that recognizes an epitope at the extreme PICALM carboxyl terminus that is found in all *PICALM* isoforms (see below). Robust PICALM expression was observed in microvessels in both non-AD and AD brain sections (Figure 1). Consistent with other reports, we also observed less robust PICALM immunostaining in other cell types that have been identified as neurons and glia [19], [20].

To elucidate the impact of the primary AD-associated SNP rs3851179 on *PICALM*, we chose a three-tiered approach. First, we evaluated whether a non-synonymous *PICALM* SNP was in linkage disequilibrium with rs3851179. The rs3851179 minor allele frequency in European Americans is 35%. According to the Exome Variant Server (http://evs.gs.washington.edu/EVS/), there are no non-synonymous *PICALM* SNPs with a minor allele frequency above 0.2% [21]. Hence, the rs3851179 association with AD is not likely to be explained by a non-synonymous *PICALM* SNP.

**Figure 2:**
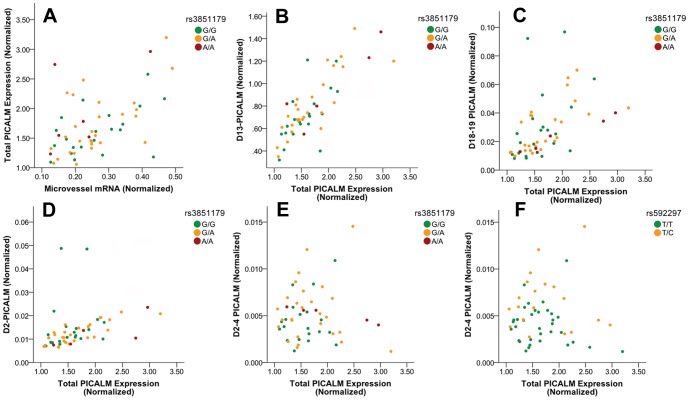
Quantitative analysis of *PICALM* isoform expression. The indicated mRNAs or isoforms was quantified by qPCR and compared relative to the AD-associated SNP rs3851179 (A–E) or rs592297, an exon 5 SNP (F).

The second tier of our approach to elucidate SNP action was to evaluate the extent that *PICALM* expression correlated with rs3851179 genotype and/or AD status. To this end, total *PICALM* expression was quantified in 52 brain samples by using qPCR and primers corresponding to sequences within exons 9 and 10 which are constitutively present (see below). *PICALM* copy number was normalized to the geometric mean of two housekeeping genes, *RPL32* and *eIF4H* [13], [14]. Inspection of the results supports that total *PICALM* expression correlated positively with microvessel mRNA expression (Figure 2A). To evaluate the statistical correlation between *PICALM* expression and relevant indices, we analyzed *PICALM* expression relative to AD status, rs3851179 genotype, and several cell-type specific mRNAs. Linear regression analysis found an overall significant model (adjusted R^2^ = 0.46) with a significant correlation between *PICALM* and rs3851179 as well as cell type markers but not AD (Table 2). Rs3851179, *GFAP* and microvessel mRNA correlated positively with total *PICALM* expression, whereas *SYP* showed negative correlation. The AD-protective, minor rs3851179A allele was associated with increased total *PICALM* expression.

**Table 2:** Multivariate Linear Regression Analysis of Total *PICALM* and Isoforms.

	Standardized Beta Coefficients			p-value
**Model: Total PICALM Expression (Adj r^2^ = 0.46)**				
AD Status		−0.05		0.66
Rs3851179		0.298		6.9×10^−3^
Microvessel mRNA		0.387		8.6×10^−4^
SYP		−0.455		1.2×10^−4^
GFAP		0.313		0.01
**Model: D13-PICALM (Adj r^2^ = 0.54)**				
AD Status		−0.304		4.9×10^−3^
Rs3851179		0.268		8.1×10^−3^
Microvessel mRNA		0.302		4.0×10^−3^
SYP		−0.519		4.2×10^−6^
GFAP		0.513		1.8×10^−5^
**Model: D18–19 PICALM (Adj r^2^ = 0.21)**				
AD Status		0.011		0.94
Rs3851179		−0.036		0.78
Microvessel mRNA		0.255		0.06
SYP		−0.36		8.0×10^−3^
GFAP		0.312		0.03
			
**Model: D2-PICALM (Adj r^2^ = 0.41)**				
AD Status		0.238		0.05
Rs3851179		0.084		0.46
Microvessel mRNA		0.287		0.02
SYP		−0.429		1.0×10^−3^
GFAP		0.24		0.06
			
**Model: D2–4 PICALM (Adj r^2^ = 0.10)**				
AD Status		−0.322		0.03
Rs3851179		0.114		0.40
Microvessel mRNA		0.002		0.99
SYP		0.233		0.10
GFAP		−0.041		0.79
			
**Model: D2–4 PICALM (Adj r^2^ = 0.24)**				
AD Status		−0.245		0.08
Rs592297		0.384		4.0×10^−3^
Microvessel mRNA		0.004		0.97
SYP		0.243		0.06
GFAP		0.001		0.99

Total *PICALM, D13-PICALM, D18–19 PICALM, D2-PICALM*, and *D2-4 PICALM* expression was analyzed as a function of AD, rs3851179 and microvessel mRNA, *SYP* and *GFAP* content by using a linear regression model. *D2-4 PICALM* was also analyzed as a function of rs592297, along with AD, microvessel mRNA, *SYP* and *GFAP*. Adj: Adjusted

**Figure 3:**
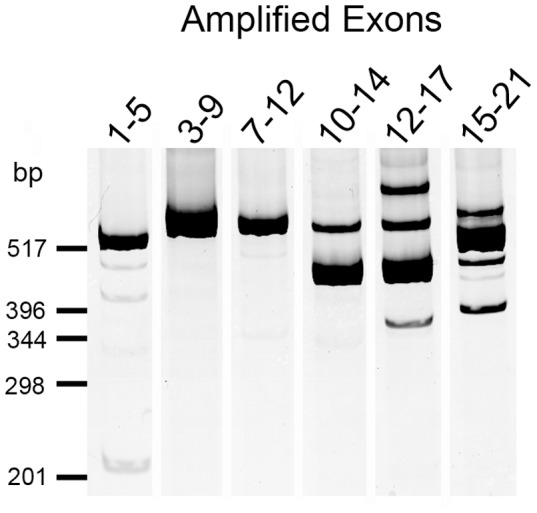
*PICALM* splice patterns in human brain. PCR amplification across the indicated exons was performed on cDNA pooled from AD and non-AD brain samples. The products were separated by polyacrylamide gel electrophoresis and visualized by SYBR-Gold fluorescence. Single PCR products from amplifications between exon 3–9 and 7–12 indicate that individual exons between 5–11 are included with high efficiency. The presence of multiple products in other lanes represents inefficiently spliced exons as confirmed in Table 3.

The third tier of our approach to determine possible SNP function was to evaluate the extent that a *PICALM* splice variant was associated with rs3851179 genotype and/or AD status. We began by identifying *PICALM* splice variants present in human brain. PCR was performed by using a series of primer pairs that flank *PICALM* internal exons, e.g., primers corresponding to exons 1 and 5 were used to evaluate whether exons 2, 3 or 4 were variably spliced. This study found that multiple *PICALM* exons were inefficiently spliced (Figure 3). Sequencing of the exon 1–5 amplicons found that most *PICALM* isoforms contained exons 2, 3 and 4 while apparently rare isoforms lacked exon 2 or exons 2–4. Amplifying from exon 3 to exon 9, and exon 7 to exon 12 showed that exons 5–11 were consistently present (Figure 3). This supports the use of primers corresponding to exons 9 and 10 for qPCR for total *PICALM*. Amplification reactions between exons 10–21 overall found multiple *PICALM* isoforms. These isoforms were not sufficiently resolved by polyacrylamide gel electrophoresis to allow sequencing of individual gel-purified products. To overcome this issue, *PICALM* from exon 12 to exon 20 was PCR-amplified, and the PCR products cloned and sequenced. To gain an initial evaluation of whether rs3851179 may be associated with *PICALM* splice variants, we analyzed RNA from three rs3851179 G/G and three rs3851179 A/A homozygous individuals. This effort revealed that exons 13, 14, 18 and 19 were inefficiently spliced. The most common *PICALM* variant lacked exon 13 and contained each of the other exons from 12 to 20 (Table 3). Other common variants contained each exon from 12–20, or lacked exon 13 and the initial 15 bp of exon 15, or lacked both exon 13 and 18. A comparison of the abundance of each isoform in rs3851179G/G versus rs3851179A/A individuals did not reveal striking differences (Table 3). Overall, we interpret these data as indicating that multiple *PICALM* exons are variably spliced. Although these isoforms were not associated with rs3851179 in this semi-quantitative assay, their abundance warranted a more quantitative evaluation.

**Table 3:** Semi-Quantitative *PICALM* Isoform Analysis.

	Rs3851179 Genotype	
*PICALM* Isoforms (exons 12–20)	AA (% Total Clones)	GG (% Total Clones)
D13	45.4±5.3	44.0±9.0
D13, 14	1.9±1.6	1.7±1.6
Dp15	1.8±2.2	4.5±4.4
D13, p15	15.3±0.5	16.4±3.2
D13, 18	9.2±4.3	9.3±1.5
D13, p15, 18	5.9±1.8	4.2±0.7
D18	2.8±2.1	3.1±1.8
D13, 18, 19	2.6±2.6	0.9±0.4
D13, p15, 18, 19	3.7±3.3	0.4±0.4
Full Length (12–20)	7.2±3.0	12.6±6.5

Complementary DNA from three rs3851179A/A and three G/G samples was amplified and the PCR amplicons cloned. A total of 847 random clones were then sequenced. This table shows the frequencies of each isoform (mean ± SD), noting that “D” indicates that an exon is missing while “p” designates a partial exon deletion, i.e., p15 refers to clones lacking first 15 bp of exon 15. Additional isoforms with an average frequency of less than 1% are not included, including rare isoforms that lacked the first 21 bp of exon 13.

For quantitation, we initially focused on exon 13 because (i) this exon is commonly skipped and (ii) this exon encodes the DPF peptide motif that contributes to PICALM binding to AP2 [22]. We quantified *PICALM* lacking exon 13 (*D13-PICALM*) by using qPCR primers corresponding to sequences within exon 11 and the exon 12- exon 14 junction (Table 1). *D13-PICALM* correlated well with total *PICALM* expression and constituted about 40% of total transcript (Figure 2B). *D13-PICALM* expression was analyzed as a function of rs3851179, AD status, and several cell-type specific mRNAs. The expression of *D13-PICALM* correlated with rs3851179, AD status, as well as the cell-type specific mRNAs (adjusted R^2^ = 0.54, Table 2). The minor rs38555179A allele and the absence of AD correlated with increased *D13-PICALM* expression.

To evaluate whether rs3851179 was associated with *D13-PICALM* independently of the SNP association with total *PICALM* expression, we analyzed *D13-PICALM* expression as a function of rs3851179, AD status and total *PICALM* expression. With this analysis, we found that *D13-PICALM* was associated with AD status and total *PICALM*, but not rs3851179. Hence, *D13-PICALM* expression is associated with rs3851179 only because total *PICALM* expression is associated with rs3851179.

We next analyzed *PICALM* splice variants that lacked exons 18 and 19 (*D18–19 PICALM*), noting that the PICALM carboxyl region that includes amino acids encoded by exon 18 and 19 is critical for PICALM function [23]. This qPCR assay used forward and reverse primers that recognized exon 17 and the exon 17 - exon 20 junction, respectively (Table 1). We found that *D18–19 PICALM* represented 1–2% of total *PICALM* expression (Figure 2C) and correlated with neuronal and astrocyte content but not rs3851179 (Table 2).

We next quantified isoforms that lack exon 2 (*D2-PICALM*). This isoform is expected to not encode a functional protein because the loss of exon 2 introduces a codon frameshift with a premature stop codon in exon 3. Exon 2 encodes a portion of the ANTH domain that binds PIP_2_ on the plasma membrane during the initial stage of clathrin-coated pit formation [24], We found that *D2-PICALM* was typically rare, representing less than 1% of total *PICALM* expression (Figure 2D). However, two samples showed increased *D2-PICALM* expression, ranging as high as 3.6%. The reason underlying the higher *D2-PICALM* in these individuals was unclear; these individuals both had AD, they differ in sex (one female and one male), and had a post-mortem interval similar to the other samples (2.4–4.0 hours). When these outlier samples were excluded from analysis, *D2-PICALM* was significantly associated with microvessel and neuronal content, as well as AD status but not rs3851179 genotype (adjusted R^2^ = 0.41, Table 2). *D2-PICALM* was increased in AD individuals.

We also identified a *PICALM* isoform lacking exons 2–4 (*D2-4 PICALM*). The *D2-4 PICALM* isoform was also present at low levels, with an average of 0.28±0.15% (mean ± S.E.) of total *PICALM* expression (Figure 2E). Expression of *D2-4 PICALM* was associated with AD but not with rs3851179 or microvessel content, suggesting that this variant is not expressed in microvessels (Table 2). Interestingly, Schnetz-Boutaud et al have reported that an exon 5 SNP rs592297 is in linkage disequilibrium with rs3851179 (D′ = 1, r^2^ = 0.34) and proposed that rs592297 modulates the activity of an exon splicing enhancer [25]. Therefore we evaluated whether rs592297 was associated with *D2-4 PICALM* expression. We found that rs592297 associated with the *D2-4 PICALM* (Figure 2F, Table 2). Hence, higher *D2-4 PICALM* expression is associated with the rs592297C minor allele. The percentage of *PICALM* expressed as *D2-4 PICALM* was quite low but was increased from 0.23±0.11% in rs592297 major allele homozygous samples to 0.36±0.17% in samples with the rs592297 minor allele (Figure 2F, p = 0.004). Although we and others have not examined the association of this SNP with AD directly, based on the linkage between rs592297 and rs3851179, the minor rs592297C allele is likely to associated with increased AD risk [25].

## Discussion

The primary findings of this paper are (i) multiple *PICALM* isoforms are expressed in human brain, (ii) consistent with immunohistochemistry results that PICALM is commonly found in microvessels, expression of total *PICALM* and the abundant *D13-PICALM* is positively correlated with the expression of microvessel mRNAs, (iii) total *PICALM* expression correlates modestly with the AD-associated SNP rs3851179, (iv) *D2-4 PICALM* was associated with AD status and an exon five SNP, rs592297, which is in linkage disequilibrium with rs3851179 (r^2^ = 0.34). However, *D2-4 PICALM* was a rare isoform, suggesting that this association is not responsible for the SNP association with AD, and (v) two additional rare *PICALM* isoforms, *D18-19 PICALM* and *D2-PICALM* were variably associated with AD and cell-specific mRNAs. Overall, we interpret our results as suggesting that multiple PICALM isoforms are expressed in the brain, and that correcting for cell-specific mRNAs allows the discernment that the AD-protective allele of rs3851179 is associated with increased *PICALM* expression.

Immunostaining showed abundant PICALM expression in microvessels. Consistent with this observation, total *PICALM* expression correlated with *CD31* and *vWF* expression, genes highly expressed in endothelial cells [17], [18]. Hence, our statistical model for *PICALM* expression included the geometric mean of these microvessel mRNAs, well as *SYP* and *GFAP*. When we analyzed *PICALM* expression in this fashion, *PICALM* expression correlated with rs3851179 genotype. Indeed, inclusion of the expression of these cell-type specific mRNA is the primary difference between our study which detected an association between PICALM expression and rs3851179 and prior studies that did not discern this association [26], [27]. The modest association that we observed may reflect that rs3851179 is not a functional SNP but rather is in linkage disequilibrium with SNP(s) that directly modulate *PICALM* expression. Rs3851179 is unlikely to be a directly functional SNP since its well removed from *PICALM* at 80 kbp upstream and does not alter a transcription factor binding site as predicted by ENCODE [28]. Hence, we speculate that another SNP, more proximal to *PICALM*, is the functional SNP and is in moderate linkage disequilibrium with rs3851179.

Variation in *PICALM* expression associated with rs3851179 genotype may have several effects. At the cellular level, PICALM mediates clathrin-coated-pit endocytosis; the amino-terminus of PICALM binds phosphatidyl-inositol 4,5 bisphosphate (PIP_2_), while the central portion binds adaptor protein-2 (AP-2) and the carboxyl terminus binds clathrin [22], [29]. Reducing PICALM expression by siRNA leads to altered size and shape of the clathrin-coated pit [22], [30]. Since the AD-protective allele of rs3851179 correlates with increased PICALM expression, we considered several mechanisms whereby PICALM may modulate AD risk. First, PICALM expression modulates APP metabolism *in vitro* [28]. Decreased PICALM expression leads to increased APP at the cell surface while increased PICALM expression leads to increased APP internalization. Since APP is metabolized in a non-amyloidogenic pathway at the cell surface but in an amyloidogenic pathway in endosomes, the effects of PICALM on APP localization lead to altered Aβ levels: PICALM knockdown reduces Aβ while PICALM overexpression increases Aβ [31]. This pathway is not consistent with our finding that the protective rs3851179 allele increases PICALM expression. A second pathway whereby altered PICALM may alter AD risk recognizes that altered PICALM expression modulates cell surface proteins in a protein-specific fashion. For example, decreased PICALM leads to increased GluR2 which may promote excitotoxicity [32]; the protective rs3851179 allele that increases PICALM expression may reduce AD risk by reducing excitotoxicity. Increased PICALM also leads to increased cell surface transferrin and EGFR [22], [30], [32], [33]. Consistent with a critical role for PICALM in iron homeostasis, PICALM-deficient mice suffer from severe anemia and poor erythroid development and, at the cellular level, show reduced transferrin uptake; iron supplementation ameliorates some aspects of PICALM deletion [23]. Recognizing that PICALM was robustly expressed in microvessels and that PICALM expression correlated positively with microvessel mRNAs, we speculate that increased PICALM may be AD-protective by facilitating Aβ clearance across the blood brain barrier [34]. Overall, altered *PICALM* levels may modulate AD risk by multiple mechanisms and is the subject of ongoing investigation.

Multiple *PICALM* exons were spliced inefficiently in human brain. Isoforms lacking many of these exons are likely to encode PICALM with altered function. Isoforms lacking exon 13 were especially common. Since a critical AP-2 binding DPF peptide motif is encoded by exon 13, the loss of exon 13 is expected to reduce AP-2 binding [22]. Loss of this DPF motif may be compensated by the DIF and/or FESVF motifs encoded within exons 12 and 14, respectively [22]. Isoforms lacking exons 13 and 14 were also detected that would lack both the DPF and FESVF motifs and would be expected to have particularly low AP-2 binding. Rare isoforms also showed an absence of exons 2 or 2–4. Since exon 2 is 143 bp, isoforms lacking exon 2 undergo a codon frameshift such that *D2-PICALM* and *D2-4-PICALM* are predicted to encode only an amino terminal PICALM fragment. Since the exon 5 SNP, rs592297, was associated with exon 2 splicing, we sought to evaluate whether this SNP was associated with AD. Although rs592297 was not available in data from Naj et al, rs1237230 is highly linked with rs592297 (r^2^ = 0.95 in Europeans, (http://www.broadinstitute.org/mpg/snap/ldsearch.php) and is present in this dataset. Rs1237230 was modestly associated with AD (p = 0.018), relative to rs3851179 (p = 0.00015) [5]. Hence, rs592297 does not appear to be robustly associated with AD risk relative to the primary *PICALM* SNP. Although rs592297 may be a functional SNP in modulating exon 2–4 splicing, the modest proportion of *PICALM* present in this isoform may mitigate the SNP effects on overall PICALM function.

In summary, our primary findings are that multiple *PICALM* isoforms are expressed in human brain, with prominent presence in microvessels, and that overall *PICALM* expression is correlated with the AD SNP rs3851179. Rare *PICALM* isoforms are associated with AD status and/or rs592297, a SNP that is in moderate linkage disequilibrium with rs3851179. The rarity of these isoforms and their lack of association with rs3851179 suggest they are unlikely to contribute to AD risk. Since *D13-PICALM* is the most abundant PICALM isoform, future studies of PICALM function may wish to evaluate this isoform.
